# Clinical aspects of a phase I trial of 5,6-dimethylxanthenone-4-acetic acid (DMXAA), a novel antivascular agent

**DOI:** 10.1038/sj.bjc.6600992

**Published:** 2003-06-10

**Authors:** M B Jameson, P I Thompson, B C Baguley, B D Evans, V J Harvey, D J Porter, M R McCrystal, M Small, K Bellenger, L Gumbrell, G W Halbert, P Kestell

**Affiliations:** 1 Department of Clinical Oncology, Auckland Hospital, Private Bag 92024, Auckland, New Zealand; 2 Auckland Cancer Society Research Centre, University of Auckland, Private Bag 92019, Auckland, NZ; 3 Drug Development Office of Cancer Research UK, 10 Cambridge Tce, London NW1 4JL, UK; 4 Cancer Research UK Formulation Unit, University of Strathclyde, 204 George St, Glasgow G1 1XW, UK

**Keywords:** DMXAA, antivascular, phase I clinical trial

## Abstract

The antitumour action of 5,6-dimethylxanthenone-4-acetic acid (DMXAA) is mediated through tumour-selective antivascular effects and cytokine induction. This clinical phase I trial was conducted to examine its toxicity, maximum tolerated dose, pharmacokinetics (PK) and pharmacodynamics (PD). A secondary objective was to assess its antitumour efficacy. DMXAA was administered every 3 weeks as a 20-min i.v. infusion. Dose escalation initially followed a modified Fibonacci schema but was also guided by PK and toxicity. A total of 63 patients received 161 courses of DMXAA over 19 dose levels ranging from 6 to 4900 mg m^−2^. DMXAA was well tolerated at lower doses and no drug-related myelosuppression was seen. Rapidly reversible dose-limiting toxicities were observed at 4900 mg m^−2^, including confusion, tremor, slurred speech, visual disturbance, anxiety, urinary incontinence and possible left ventricular failure. Transient prolongation of the corrected cardiac QT interval was seen in 13 patients evaluated at doses of 2000 mg m^−2^ and above. A patient with metastatic cervical carcinoma achieved an unconfirmed partial response at 1100 mg m^−2^, progressing after eight courses. The results of PK and PD studies are reported separately. DMXAA has antitumour activity at well-tolerated doses.

Targeting tumour vasculature as a treatment for cancer is the subject of much research, with most effort currently directed at angiogenesis inhibitors. Yet therapies that interrupt existing tumour vasculature and result in haemorrhagic necrosis of tumours have been investigated for over a century and were mostly associated with bacterial infections or their toxins (Chaplin and Dougherty, 1999). William Coley, who pursued the method avidly from 1891 (Council on Pharmacy and Chemistry, 1934), achieved significant cure rates, especially in sarcoma and lymphoma, using various bacterial toxins both for advanced disease and as adjuvant therapy (Coley-Nauts *et al*, 1953). Coley's toxins were abandoned in favour of radiotherapy, but more recent clinical studies with bacterial endotoxins have shown modest antitumour activity, although systemic toxicity remains dose-limiting (Otto *et al*, 1996; DeVore *et al*, 1997).

In 1947, investigators at the National Cancer Institute observed that a toxin from the bacterium *Serratia marcescens* acutely and irreversibly reduced blood flow to sarcomas in mice, while blood flow to muscle recovered within 18 h (Algire *et al*, 1947). The induction of tumour necrosis factor (TNF) by bacterial toxins was later shown to be the cause of haemorrhagic necrosis in tumours (Carswell *et al*, 1975; Old, 1985). Clinical trials of recombinant human TNF were unable to deliver therapeutic doses systemically because of toxicity (Spriggs and Yates, 1992), but TNF is highly effective in conjunction with chemotherapy in isolated limb perfusion for melanoma and sarcoma, where it has been shown to have selective tumour vascular effects (Lejeune *et al*, 1998; Ruegg *et al*, 1998; Eggermont and ten Hagen, 2001).

Nonbacterial, tumour vascular-targeting agents could have advantages over bacterial products in terms of toxicity and pharmacology. One such agent, flavone acetic acid (FAA), showed remarkable anticancer activity in murine models (O'Dwyer *et al*, 1987) but its activity was minimal in clinical trials, although these were conducted when it was thought to be directly cytotoxic and its indirect mechanisms of antitumour action were poorly understood (Kerr and Kaye, 1989; Bibby, 1991). Further preclinical studies at the Auckland Cancer Society Research Centre (ACSRC) showed that FAA induced TNF production, acute tumour vascular collapse, haemorrhagic necrosis of tumours and enhanced activity of immune effector cells (Ching and Baguley, 1987; Smith *et al*, 1987; Finlay *et al*, 1988; Zwi *et al*, 1989, 1992) (Zwi *et al*, 1990 ID: 286). Few appropriate pharmacodynamic (PD) studies were performed in the clinical trials of FAA, so it is not known whether it has significant antivascular or immunological activity in humans. However, *in vitro* studies suggested a species difference in antitumour activity, because FAA induced TNF in murine, but not human, peripheral blood mononuclear cells (Ching *et al*, 1994; Philpott *et al*, 1997).

5,6-Dimethylxanthenone-4-acetic acid (DMXAA), a new analogue of FAA developed in the ACSRC, showed greater activity and 12-fold higher dose-potency than FAA in murine tumour models (Rewcastle *et al*, 1991) and appeared to overcome the species difference in *in vitro* TNF production (Ching *et al*, 1994; Philpott *et al*, 1997). Dose-limiting toxicity in mice was consistent with hypotension, and was not strictly related to TNF production (Philpott *et al*, 1995). While DMXAA proceeded into clinical development because of this favourable profile, further studies in animal tumour models have demonstrated synergistic interactions between DMXAA with radiotherapy (Wilson *et al*, 1998), chemotherapy (particularly taxanes) (Pruijn *et al*, 1997; Horsman *et al*, 1999; Wilson and Baguley 2000; Siim *et al*, 2002), bioreductive cytotoxic drugs (Cliffe *et al*, 1994; Lash *et al*, 1998), radioimmunotherapy (Pedley *et al*, 1996), antibody-directed enzyme prodrug therapy (ADEPT) (Pedley *et al*, 1999), thalidomide (Cao *et al*, 1999) and immunotherapy (Kanwar *et al*, 2001).

Cancer Research UK selected DMXAA for two parallel phase I trials with different schedules, one in the UK using a weekly administration schedule (at Mt Vernon Hospital, Northwood, and Bradford Royal Infirmary, Bradford) and the other in Auckland, NZ, dosing every 3 weeks. The objectives of the NZ study were to determine the toxicity of DMXAA, its maximum tolerated dose (MTD), pharmacokinetics (PK), selected PD end points and, as a secondary objective, antitumour efficacy. A protocol amendment permitted evaluation of changes in tumour blood flow by dynamic magnetic resonance imaging (MRI) and the findings (including both UK and NZ trial patients) have recently been published (Galbraith *et al*, 2002). This report focuses on clinical aspects of the NZ trial; PD and PK aspects, due to their complexity, will be reported separately.

## MATERIALS AND METHODS

### Patients

Eligibility criteria included: patients ⩾18 years with histologically or cytologically proven cancer refractory or not amenable to conventional therapy; WHO performance status of 0–2; life expectancy >3 months; haemoglobin ⩾9 g dl^−1^, WBC ⩾3 × 10^9^ l^−1^, platelets ⩾100 × 10^9^ l^−1^; creatinine ⩽130 *μ*mol l^−1^; bilirubin within normal limits, ALT and alkaline phosphatase <2 × upper limit of normal unless due to liver and/or bone metastases; coagulation (international normalised ratio (INR) and activated partial thromboplastin time (APTT)) within normal limits; not pregnant or lactating (adequate contraception if capable of child-bearing); no anticancer therapy within 4 weeks (6 weeks for nitrosoureas and mitomycin C); no other serious medical conditions, uncontrolled infection or serious infection within 28 days; no glucocorticoid treatment in excess of physiological replacement doses within 2 weeks; and no concurrent malignancies with the exception of cone-biopsied cervical intraepithelial neoplasia and adequately treated basal or squamous cell carcinoma of the skin. All patients gave written informed consent and the regional ethics committee approved the study.

### Drug administration

DMXAA (Figure 1

**Figure 1:**
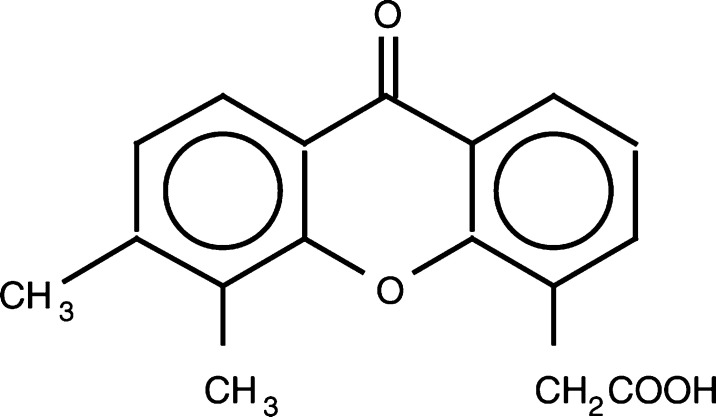
Structure of DMXAA.

) was formulated as a 20 mg ml^−1^ solution of the sodium salt in 0.1 M phosphate buffer, pH 7.7 and stored in amber glass ampoules at room temperature. Exposure of DMXAA to direct sunlight was avoided to prevent decarboxylation and precipitation (Rewcastle *et al*, 1990). A more concentrated formulation (100 mg ml^−1^ in 0.02 M phosphate buffer, pH 7.9) was developed in order to reduce infusion volumes at higher doses. The drug was administered every 3 weeks as a 20-min i.v. infusion (made up to a minimum volume of 100 ml with normal saline) using Imed 960A or Gemini PC-2 infusion pumps (Alaris Medical Systems, San Diego, CA, USA). Patients could receive a maximum of six courses if static disease was seen, but if responding they could continue to maximum response plus two courses up to a maximum of 12 courses. No intrapatient dose escalation was permitted, but when a partial response was observed a protocol amendment was introduced to allow patients treated at lower doses to re-enter the trial at a higher dose level (if still eligible) to gain the opportunity of therapeutic benefit.

### Dose escalation

The starting dose of 6 mg m^−2^ was one-tenth of the LD_10_ in mice. Dose escalation was planned to follow a modified Fibonacci scheme, but could be modified by PK data and the clinical judgement of the investigators and a protocol amendment permitted results from the parallel UK phase I study to influence dose escalation. Three patients were planned at each dose level and three additional patients were to be entered if any of the following toxicities occurred: neutrophil nadir <1.0 × 10^9^ l^−1^; platelet nadir <75 × 10^9^ l^−1^; grade 2 neurologic or cardiac toxicity or an increase of one grade of peripheral neuropathy if present at baseline; grade 3 vomiting not responding to symptomatic treatment or other grade 3 nonhaematological toxicity. The MTD was defined as the dose below that at which two of three patients suffered a dose-limiting toxicity (DLT) due to the drug (neutrophil nadir <0.5 × 10^9^ l^−1^ or platelet nadir <25 × 10^9^ l^−1^ or Grade 2 neurotoxicity or Grade 3 other nonhaematological toxicity (excluding alopecia and nausea)). When DLT was reached, an additional three patients were to be treated at the previous dose level to determine the recommended dose for phase II trials.

### Patient assessments

Prior to treatment and weekly while on trial each patient had a history taken, clinical examination and blood tests including complete blood count (CBC) and differential, INR, APTT, chemistry and urinalysis. Complete blood count was repeated 24 and 72 h after course 1 and 24 h after course 4. International normalised ratio and APTT were repeated 4 h after DMXAA infusion on courses 1 and 4. Blood pressure and heart rate were monitored from 30 min before each infusion to 6 h afterwards. On course 1 (and on course 2 in one patient at each dose level) patients were hospitalised for 24 h to monitor toxicity and PK. Later in the trial, 24-h urine collections were made immediately prior to and after DMXAA administration, in order to evaluate changes in creatinine clearance. The National Cancer Institute of Canada Clinical Trials Group (NCIC-CTG) Expanded Common Toxicity Criteria (CTC) was used to assess adverse events.

An electrocardiograph (ECG) was performed prior to trial entry. When transient prolongation of the cardiac corrected QT interval (QT_c_) was noticed in a patient after treatment with DMXAA at 2000 mg m^−2^, subsequent patients had serial ECGs (in two patients) or ambulatory digital Holter ECG monitoring (in 12 patients) to evaluate acute changes in the QT_c_. The QT_c_ was calculated using Bazett's formula (QT_c_=QT/square root of preceding R-R interval) (Kligfield *et al*, 1996) and was measured in multiple ECG traces over the first 6–24 h after DMXAA administration.

Tumour response was assessed clinically each week, by plain radiographs every 3 weeks and by computed tomography (CT) or ultrasound scans every 6 weeks. A complete response was defined as the disappearance of all disease on two assessments at least 4 weeks apart. A partial response was defined as at least 50% decrease in the sum of the products of bidimensional measurements (or the sum of maximum diameters of lesions where bidimensional measurements were not feasible) of defined measurable lesions on two assessments at least 4 weeks apart, and no lesion should have progressed and no new lesions appeared. No change (static disease) was defined as less than 50% decrease and less than 25% increase in these measurements.

## RESULTS

### Patient characteristics

Table 1

**Table 1:** Patient characteristics

**Characteristic**	**No. of patients**
Total patients	63

*Gender*	
Male	21
Female	42

*Age (years)*	
Median	55
Range	24–75

*WHO performance status*	
0	13
1	22
2	28

*Tumour primary site*	
Colon/rectum	20
Ovary	11
Melanoma	6
Lung	5
Breast	3
Cervix	3
Unknown primary	3
Pancreas	2
Oesophagus	2
Kidney	2
Soft-tissue sarcoma	2
Other	4

*Previous treatment*	
Chemotherapy	55
Radiotherapy	25
Biotherapy/hormones	11
None	1

summarises the gender, age, performance status, diagnosis and prior systemic therapy of patients enrolled onto the trial. A total of 161 courses were administered to 63 patients (60 individual patients, three of whom were re-enrolled at a higher dose level and evaluated as new patients). The median number of courses was 2 (range 1–8).

### Dose escalation

Table 2

**Table 2:** Dose escalation schedule

**DMXAA dose level (mg m^−2^)**	**No. of patients**	**No. of courses**	**Patients with DLT**
6	3	11	0
10.2	3	9	0
20.4	3	6	0
40.8	3	6	0
81.6	3	4	0
160	3	6	0
240	3	7	0
360	3	6	0
500	6	19	0
650	3	6	0
850	3	6	0
1100	3	13	0
1375	3	9	0
1650	3	8	0
2000	3	7	0
2600	3	10	0
3100	3	5	0
3700	7^a^	18	1
4900	3^a^	5	3

DLT=dose-limiting toxicity.

a One patient treated at both dose levels.

summarises the dose escalation schedule, number of courses administered and number of patients with DLT at each dose level. Where the investigators felt that specific toxicities were not of sufficient clinical concern, dose escalation continued without the dose level being expanded. One patient experienced DLT at the highest dose level and dropped one dose level for the second course, and is therefore represented twice. The dose was escalated from 6 to 4900 mg m^−2^ over 19 dose levels. The first escalation (1.7-fold) followed the planned Fibonacci series, but the values of the area under the plasma concentration–time curve (AUC) at the first dose level were much lower (1/30th) than those predicted from murine data. Therefore, the dose was doubled for subsequent escalations. A rise in plasma nitrate (one of the PD end points measured) in a patient at 160 mg m^−2^ suggested the possibility of having reached a PD threshold, and further escalations (1.2–1.5-fold) were guided by toxicity and PK from both the UK and NZ trials. Dose escalation was not restricted by nonhaematological toxicities that technically met the protocol definition for DLT but were not considered of sufficient clinical significance (such as acute onset of tremor that resolved within one hour).

### General toxicity

Toxicities of DMXAA were markedly different from those of many cytotoxic drugs, and many patients commented that it was easier to tolerate than chemotherapy. There was no significant drug-related neutropenia, thrombocytopenia or coagulopathy, and while anaemia and lymphocytopenia were common, they were judged unrelated to DMXAA in the majority of cases. Table 3

**Table 3:** Nonhaematological toxicity (drug-related)

		**Maximum grade of toxicity per patient**													
		**Nausea and/or vomiting**		**Venous pain**		**Malaise**		**Hyper- or hypotension**		**Flushing/sweating**		**Acute neuro symptoms**		**Dyspnoea**	
**Dose (mg m^−2^)**	**No. of patients**	**1–2**	**3–4**	**1–2**	**3–4**	**1–2**	**3–4**	**1–2**	**3–4**	**1–2**	**3–4**	**1–2**	**3–4**	**1–2**	**3–4**
6	3	1					1			1					
10.2	3	1					1								
20.4	3	1				2									
40.8	3														
81.6	3	1						1							1
160	3	1		1						1					
240	3	1		1											
360	3			2						1					
500	6	1		2		1		2		1					
650	3	2		1											1
850	3			2				1							
1100	3	2		3		1		3							1
1375	3			3				1				2			
1650	3	3		1		2		1				2			
2000	3	2		2				1		1		3			
2600	3		2	2		1		2		3		3			1
3100	3	3		2				2		3		3			1
3700	7	6		6				6		7		7			2
4900	3	3		3				3		3			3		1

a Almost certainly, probably or possibly related to DMXAA. Neuro=neurological.

summarises selected drug-related non-haematological toxicities, which established the MTD at 3700 mg m^−2^. At lower dose levels, this drug was generally well tolerated, with a minority of patients experiencing mild drug-related toxicity. Acute symptoms included venous discomfort with the infusion (alleviated by application of a warm pad), increased tumour pain several hours after the drug infusion and one patient briefly developed small urticarial lesions. Delayed toxicities included ‘flu-like’ symptoms (malaise, myalgia, fatigue, nausea, sweating) on days 2–4 of each course, but no mucositis or alopecia occurred. Additional acute toxicities were more prevalent with increasing dose above 1100 mg m^−2^, with onset of symptoms during or shortly after the 20-min drug infusion. They were usually mild and invariably transient, generally resolving between a few minutes and 4 h later, and included nausea and/or vomiting (for only a few minutes and not reliably prevented by ondansetron), sympathetic disturbance (sweating, warm flush, hypotension, hypertension, bradycardia or tachycardia), altered taste and neurological disturbance (visual disturbance, tremor, feeling light-headed and restless). Patients at the higher dose levels also commonly experienced a brief urge to urinate and/or defecate. One patient (treated at 1650 mg m^−2^) had pre-existing impairment of bladder control and was incontinent of urine immediately following DMXAA administration. Creatinine clearance (measured by two sequential 24-h urine collections in 12 patients) did not change following DMXAA administration at doses ranging from 1375 to 4900 mg m^−2^ (paired *t*-test, *P*=0.63).

### Neurological toxicity

At 4900 mg m^−2^, acute neurological toxicity was considered dose limiting in all three patients, even though symptoms resolved completely within 2 h. These included slurred speech (two patients), confusion and expressive dysphasia (one patient), moderate anxiety (one patient), severe visual disturbance (one patient), tremor (two patients) and urinary incontinence (one patient). Similar neurological toxicity was seen in one patient treated at 1650 mg m^−2^ on her second course only, attributed to an interaction between DMXAA and isocarboxazid, a monoamine oxidase inhibitor. The features were consistent with the ‘serotonin syndrome’ (Sporer, 1995) and included difficulty concentrating, drowsiness, generalised tremor, occasional myoclonic jerks, hyper-reflexia, bradycardia and hypertension, all resolving within 6 h.

Visual disturbance was first reported at 1375 mg m^−2^ and symptoms became more prevalent and intense with increasing dose. Symptoms at lower dose levels included altered colour vision, blurring (without impairment of visual acuity) and mild photophobia. Colour vision testing revealed acute deterioration in colour discrimination, resolving within 4 h. Dose-limiting transient visual disturbance at 4900 mg m^−2^ included glittering of objects, harsh contrasts, jerky motion and strobe effects. Pattern and flash electroretinogram (ERG) showed an acute increase in latency and reduction in amplitude of certain retinal responses following DMXAA infusion with subsequent return to baseline over several hours, and a dose–response relationship was observed. A patient who received six courses at 3700 mg m^−2^ showed subclinical deterioration of pre-infusion ERGs between courses 2 and 6.

### Cardiorespiratory toxicity

**Figure 2:**
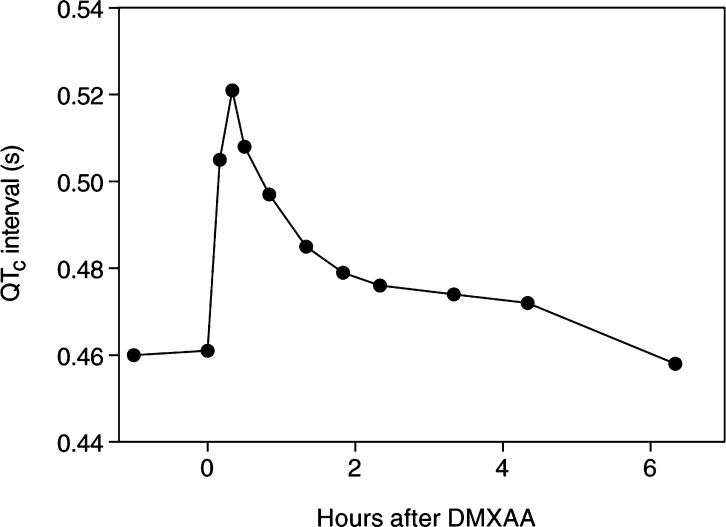
Change in corrected QT interval after DMXAA administration at 3700 mg m^−2^.

Transient prolongation of the corrected cardiac QT interval was seen in all 13 patients evaluated at doses of 2000–4900 mg m^−2^ (median prolongation 52 ms, range 38–100 ms). In six of nine patients with normal baseline QT_c_ values, QT_c_ became abnormally prolonged (>450 ms in males, >470 ms in females) after treatment with doses of DMXAA of ⩾3100 mg m^−2^ (Committee for Proprietary Medicinal Products, 1997). The maximal QT_c_ prolongation occurred during or within 15 min of completion of the drug infusion with a return to baseline generally over 4–6 h (Figure 2

). QT_c_ was prolonged beyond 500 ms in four patients with baseline values of 430, 460, 461 and 501 ms and in the first three patients, this lasted for less than 1 h. No clear dose–response relationship was observed and no ventricular tachyarrhythmia was seen.

Dyspnoea possibly related to DMXAA was documented in three patients treated at doses ⩽1100 mg m^−2^, but each patient had another more likely explanation. At higher doses, acute dyspnoea at rest occurred in four patients immediately following DMXAA infusion. In three of these patients (treated at 2600–3700 mg m^−2^), the dyspnoea after DMXAA was minimal, brief (5–25 min) and clinically insignificant. The fourth patient, with moderately severe chronic obstructive respiratory disease, became very breathless and anxious 15 min after her first course at 4900 mg m^−2^. Clinically, she had basal pulmonary inspiratory crackles but no bronchospasm. Oxygen saturation was normal on breathing air and the symptoms resolved in about 75 min. On her second course, DMXAA was reduced to 3700 mg m^−2^ (she is thus represented at two doses in Table 3) and she again developed dyspnoea, lasting for about an hour. She had basal inspiratory crackles and a third heart sound (consistent with left ventricular failure) and these signs resolved within 2 h.

### Therapeutic response

**Figure 3:**
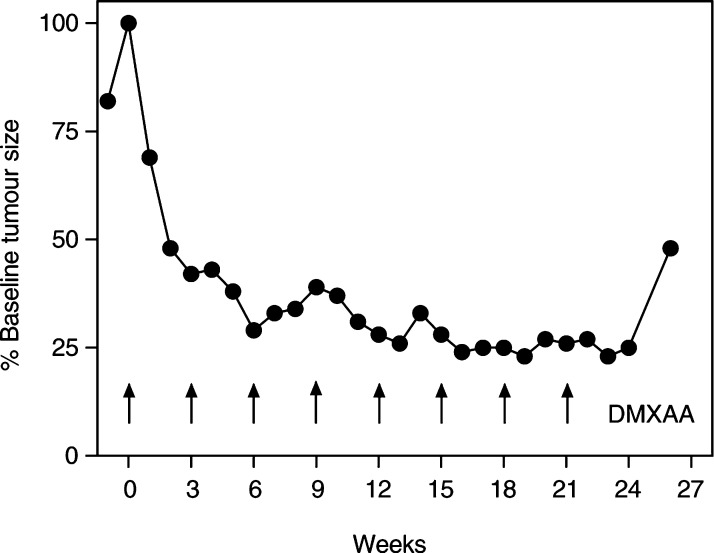
Tumour response in a patient with metastatic squamous carcinoma of cervix treated with DMXAA 1100 mg m^−2^. Tumour size was calculated as the sum of the products of bidimensional measurements of three clinically measurable metastatic neck nodes.

A total of 60 patients were evaluable for response. One partial response was seen in a patient with metastatic squamous carcinoma of cervix treated at 1100 mg m^−2^ and previously treated with bleomycin, etoposide and cisplatin chemotherapy, then carboplatin. The response was unconfirmed because two small neck nodes increased in size transiently after the third course then subsequently regressed again and overall tumour response was maintained for eight courses (Figure 3

). No change was the best response in 22 patients (35%) at dose levels from 6 to 3700 mg m^−2^ and the duration exceeded 12 weeks in five patients (8%).

## DISCUSSION

This phase I trial of DMXAA has demonstrated clinical antitumour activity and reduction in tumour blood flow at well-tolerated doses (Galbraith *et al*, 2002). The MTD was established as 3700 mg m^−2^, and higher doses produced unusual, transient toxicities including confusion, slurred speech, tremor, restlessness and visual disturbance, as well as transient autonomic changes such as sweating, tremor, changes in blood pressure and heart rate. The results of the UK trial of DMXAA are published separately (Rustin *et al*, submitted to *Br J Cancer*), but there was a high degree of concordance with our results including toxicities, PK, PD and clinical activity (an unconfirmed partial response was also seen).

Clinical trials of FAA, which is structurally related to DMXAA, showed differences between the two drugs. While the clinical toxicities of DMXAA and FAA showed some similarities (warmth, flushing, sweating, fatigue, myalgia, nausea, vomiting and visual disturbance), the DLTs of FAA were remarkably different and included hypotension, diarrhoea, flushing, asthenia and fatigue (Kerr *et al*, 1987; Weiss *et al*, 1988; Kaye *et al*, 1990; Havlin *et al*, 1991; Olver *et al*, 1992). Prolongation of bleeding time was described (Rubin *et al*, 1987) and one patient presented with haemorrhage due to immune thrombocytopenia after five doses of FAA (Davis *et al*, 1988). Bleeding time was not measured in this DMXAA trial, but no significant disturbance of INR and APTT was seen, nor was any significant thrombocytopenia observed.

A number of other agents, including serotonin, some tubulin-binding agents and arsenic trioxide, selectively inhibit tumour blood flow (Stücker *et al*, 1992; Ching *et al*, 1999; Lew *et al*, 1999). Combretastatin A-4 phosphate (CA4P), a tubulin-binding agent, has antivascular activity at doses well below the MTD in both preclinical and early clinical studies (Rustin *et al*, 1999), comparable to those reported with DMXAA (Lash *et al*, 1998; Galbraith *et al*, 2002). However, the blood flow-modifying effect of CA4P is not entirely tumour-selective (Griggs *et al*, 2001), and it appears to be reversible except at high doses (Anderson *et al*, 2000; Murata *et al*, 2001). Some CA4P toxicities resembled those of DMXAA (including flushing, nausea, vomiting, tumour pain and QT_c_ prolongation), but others (cardiac ischaemia and cerebellar ataxia) were notably different (Galbraith *et al*, 2001; Dowlati *et al*, 2002).

Some toxicities observed with DMXAA resemble the ‘serotonin syndrome’, attributed to high levels of serotonin in the central nervous system (CNS). This syndrome most commonly occurs when two drugs are taken, which can each increase CNS serotonin and includes alterations in cognition, behaviour, autonomic nervous system function and neuromuscular activity (Sporer, 1995). Supportive evidence comes from two other aspects of this trial: firstly, a patient took a monoamine oxidase inhibitor prior to treatment with DMXAA at 1650 mg m^−2^ and developed clinical features of the serotonin syndrome; and secondly an acute increase in plasma prolactin levels seen in many patients treated with DMXAA at ⩾2000 mg m^−2^ (unpublished results). This occurs following production of serotonin in the CNS (Van de Kar *et al*, 1996). If subsequent studies provide further evidence that DMXAA increases CNS serotonin release, it would be prudent to avoid administering DMXAA to patients who are receiving other drugs that increase CNS serotonin levels (Brown *et al*, 1996). The mechanism underlying the acute release of CNS serotoninis not known, but serotonin release in plasma has been observed in this study (Kestell *et al*, 2001), and is a feature of the antivascular activity of this drug in preclinical models (Baguley *et al*, 1997).

The visual toxicities of DMXAA are not a feature of the serotonin syndrome, but the blurring, colour disturbance and photophobia have similarities to those reported with sildenafil (Viagra®, Pfizer, New York, NY, USA) (Marmor and Kessler, 1999). The latter's visual toxicities are thought to be due to inhibition of phosphodiesterase type 6 (PDE6), which exists exclusively in the retina and is responsible for modulating the transduction cascade of the photoreceptor response to light. Recent data have shown that DMXAA inhibited PDE6 *in vitro* at pharmacologically relevant concentrations (e.g. 50 *μ*M) (personal communication, L Kelland, Antisoma plc). It is reassuring in this regard that no long-term retinal sequela of sildenafil administration is known, including data from retinal histologic studies in dogs dosed with 65 times the maximum recommended human dose daily for 12 months (Wallis *et al*, 1998; Marmor and Kessler, 1999).

The significance of the observed QT_c_ prolongation in these preliminary data is uncertain given its brevity, its possible relation to autonomic changes caused by the infusion and the population of patients potentially being treated with this agent. Moreover, the use of *ad hoc* heart rate correction formulae (such as Bazett's) may bias the result (Aytemir *et al*, 1999). Therefore the QT_c_ results in this trial must be regarded as indicative only. However, given the concern that QT interval prolongation may predispose to ventricular tachycardia (Committee for Proprietary Medicinal Products, 1997), it will be important to determine the dose–response relationship of QT_c_ prolongation before deciding on the dose of DMXAA for phase II and combination studies.

In conclusion, DMXAA is well tolerated over a wide dose range and has clinical and biological features distinct from those of other antivascular drugs in clinical development. Further clinical trials with this agent are clearly warranted, particularly in combination with other treatment modalities where synergistic interactions are observed in animal tumour models.
